# Patients with primary biliary cholangitis and fatigue present with depressive symptoms and selected cognitive deficits, but with normal attention performance and brain structure

**DOI:** 10.1371/journal.pone.0190005

**Published:** 2018-01-10

**Authors:** Roman Zenouzi, Janina von der Gablentz, Marcus Heldmann, Martin Göttlich, Christina Weiler-Normann, Marcial Sebode, Hanno Ehlken, Johannes Hartl, Anja Fellbrich, Susanne Siemonsen, Christoph Schramm, Thomas F. Münte, Ansgar W. Lohse

**Affiliations:** 1 1st Department of Medicine, University Medical Center Hamburg-Eppendorf, Hamburg, Germany; 2 Department of Neurology, University of Lübeck, Lübeck, Germany; 3 Department of Interdisciplinary Endoscopy, University Medical Center Hamburg-Eppendorf, Hamburg, Germany; 4 Department of Diagnostic and Interventional Neuroradiology, University Medical Center Hamburg-Eppendorf, Hamburg, Germany; University of Navarra School of Medicine and Center for Applied Medical Research (CIMA), SPAIN

## Abstract

**Background:**

In primary biliary cholangitis (PBC) fatigue is a major clinical challenge of unknown etiology. By demonstrating that fatigue in PBC is associated with an impaired cognitive performance, previous studies have pointed out the possibility of brain abnormalities underlying fatigue in PBC. Whether structural brain changes are present in PBC patients with fatigue, however, is unclear. To evaluate the role of structural brain abnormalities in PBC patients severely affected from fatigue we, therefore, performed a case-control cerebral magnetic resonance imaging (cMRI) study and correlated changes of white and grey brain matter with the cognitive and attention performance.

**Methods:**

20 female patients with PBC and 20 female age-matched controls were examined in this study. The assessment of fatigue, psychological symptoms, cognitive and attention performance included clinical questionnaires, established cognition tests and a computerized test battery of attention performance. T1-weighted cMRI and diffusion tensor imaging (DTI) scans were acquired with a 3 Tesla scanner. Structural brain alterations were investigated with voxel-based morphometry (VBM) and DTI analyses. Results were correlated to the cognitive and attention performance.

**Results:**

Compared to healthy controls, PBC patients had significantly higher levels of fatigue and associated psychological symptoms. Except for an impairment of verbal fluency, no cognitive or attention deficits were found in the PBC cohort. The VBM and DTI analyses revealed neither major structural brain abnormalities in the PBC cohort nor correlations with the cognitive and attention performance.

**Conclusions:**

Despite the high burden of fatigue and selected cognitive deficits, the attention performance of PBC patients appears to be comparable to healthy people. As structural brain alterations do not seem to be present in PBC patients with fatigue, fatigue in PBC must be regarded as purely functional. Future studies should evaluate, whether functional brain changes underlie fatigue in PBC.

## Introduction

Primary biliary cholangitis (PBC) is a chronic autoimmune liver disease characterized by a non-suppurative destructive cholangitis of the small intrahepatic bile ducts. The disease presents usually many years prior to the development of end-stage liver disease, and its natural history of liver cirrhosis can often be prevented with timely diagnosis and sufficient treatment [1].

While in most cases the liver manifestation of the disease is amenable to treatment, the majority of PBC patients suffer from a number of extrahepatic symptoms, e.g. Sicca-syndrome, arthralgia and, most of all, fatigue. Fatigue is a complex symptom characterized by the feeling of exhaustion, lethargy, and discomfort and affects approximately 50% of PBC patients. Despite its major impact on patients´ quality of life as well as survival, treatment options for fatigue, however, are very limited, resulting in frustration in both, patients and physicians [2–4].

Searching for treatment options for fatigue requires a better understanding of its pathophysiology. Interestingly, previous studies have pointed out that fatigue in PBC is independent from the hepatic disease activity or stage and does not substantially improve after liver transplantation [2–6]. This points towards an extrahepatic pathway underlying fatigue in PBC. Some authors have proposed changes in the central nervous system (CNS) to be responsible for fatigue in PBC [4,6]. This idea mainly originates from reports on CNS changes in bile duct resected rats [7] or cognitive dysfunction of affected patients [8,9]. A very limited number of neuroimaging studies have reported white matter or globus pallidus abnormalities in PBC patients with fatigue by using T1- and T2-weighted cerebral magnetic resonance imaging (cMRI) or magnetization transfer scans. These studies, however, were rather small, partly uncontrolled or failed to correlate CNS alterations found with the patients´ degree of fatigue [9–11].

Diffusion tensor imaging (DTI) is a MRI technique that provides unique insights into brain network connectivity by measuring the diffusive motion of tissue water. DTI particularly enables to explore the white matter structure in vivo and has thus been implemented to investigate abnormalities in numerous neurological disorders [12]. While several reports have demonstrated a relationship between the degree of white matter abnormalities and fatigue in multiple sclerosis, a neuroimmunological disorder often associated with fatigue [13], DTI data for PBC are scarce and limited to a small pilot study that recently reported altered diffusion coefficients in the thalamus of pre-cirrhotic PBC patients [14].

We hypothesized that in patients with PBC severe fatigue is associated with an impaired cognitive and attention performance as well as significant CNS alterations in form of structural brain abnormalities. We thus performed a case-control structural cMRI study with PBC patients and correlated the results to the degree of the study participants´ cognitive and attention performance.

## Materials and methods

### Human subjects

The study protocol conformed to the ethical guidelines of the 1975 Declaration of Helsinki and was approved by the local ethics committee (PV3932; Ethics Committee of the Medical Association Hamburg, Germany). After obtaining informed written consent, 20 female patients with well-defined PBC according to the practice guidelines for cholestatic liver diseases of the European Association for the Study of the Liver [1] as well as 20 healthy age-matched (±3 years) female volunteers were included. All PBC patients were outpatients of a tertiary care center in Germany (Hamburg). In order to reveal potential fatigue associated cognitive or attention deficits as well as structural brain changes in the PBC group, only PBC patients were included, who reported to be severely affected from fatigue on regular outpatient clinic visits. Nevertheless, the severity of fatigue and associated symptoms were additionally measured using established questionnaires (see experimental procedure). After study inclusion, however, no patient was excluded due to an unexpectedly low fatigue score. All participants were right handed without a history of neurological disease, contraindications for MRI and without any known fatigue-related disease other than PBC.

### Experimental procedure

For all subjects, the experimental procedure included clinical scales to assess fatigue and psychological symptoms (i), a cognitive assessment (ii), a computerized test battery of attention performance (TAP) (iii) as well as cMRI measurement (iv). For a detailed description of the experimental procedure see the supporting information (S1 Appendix).

(i) Previously described fatigue scores for PBC are not available in a validated form in German [15,16]. Therefore, the Würzburg depletion Inventory for multiple sclerosis (WEIMUS, German, Würzburger Erschöpfungsinventar für Multiple Sklerose), a validated instrument that measures fatigue in patients with multiple sclerosis and differentiates between physical and cognitive fatigue, was used for fatigue assessment [17]. Psychological symptoms potentially associated to fatigue were assessed with the “Beck Depression Inventory” (BDI) [18] and the “Symptom check list of Derogatis” (SCL-90-R) [19].

(ii) The cognitive assessment addressed working memory (digit span test, digit ordering test A and B) [20], cognitive flexibility (trailmaiking test A and B) [21–23] and verbal fluency (German, Regensburger Wortflüssigkeitstest. Subtests: lexical and semantic) [24].

(iii) To measure different components of attention a computerized test battery of attention performance (TAP, PSYTEST, Herzogenrath, Germany) was used, including the subtests alertness, divided attention (auditive and visual), vigilance and alertness in this sequence (duration: approximately 45 minutes). In every subtest the participant had to react to a stimulus on a screen as fast as possible by pressing a button. Reaction time was recorded. The subtest alertness consisted of two parts, with and without an acoustic warning signal before the stimulus presentation in order to investigate the tonic and phasic alertness and was repeated at the end of the TAP (alertness II) to detect deterioration over time. For the same reason, results from the 30 minutes subtest vigilance were recorded separately for the first and second half of the test.

(iv) Structural images were acquired on a 3 Tesla Siemens MAGNETOM Skyra scanner with a 20-Channel head coil. We recorded a T1-weighted 3D dataset with a MPRAGE sequence with a repetition time of 2400 ms, an echo time of 3.59 ms with 0.8 mm isotropic voxel and a field of view of 256 x 256. Diffusion-weighted sequences consisted of 50 axial slices with a 128 x 128 matrix, 2 mm slice thickness and 2 mm interslice gap. They were measured using a repetition time of 7200 ms, an echo time of 86 ms and an 80° flip angle.

### Statistical analysis of the clinical and experimental (non cMRI/DTI) data

Patient and experimental data (i-iii) including figure bars were described using the median value accompanied by the first (Q1) and the third quartile (Q3). For the statistical analysis a Wilcoxon matched pairs test was used as test results were irregularly distributed (D'Agostino & Pearson omnibus normality test) and a p-value <0.05 was considered to be statistically significant. To compare the deterioration of reaction time during the subtest vigilance (TAP) a two-way ANOVA was used.

### Voxel-based morphometry analysis

Voxel-based morphometry (VBM) is a technique to analyses structural changes of the brains grey matter using T1-weighted MR images. VBM analysis was carried out using Statistical Parametric Mapping 12b (SPM, http://www.fil.ion.ucl.ac.uk/spm). After preprocessing a two-sample t-test was computed as group statistic for every voxel, whereby age and intracranial volume were considered as confounding factors. With respect to the whole cohort, we additionally correlated the results of selected neuropsychological tests with the grey matter volume using a multiple regression analysis. All results were corrected for multiple testing using the false discovery rate correction and considered at a false discovery rate corrected p-value of 0.05 with xjview (https://www.nitrc.org/projects/xjview) [25]. To detect grey matter differences that may become significant at a larger sample size we considered the results also at an uncorrected p-value of 0.001, which is a common method to explore patient data. Due to an increase of the alpha error it has to be acknowledged, though, that this approach may produce false positive results.

### Diffusion tensor imaging analysis

Preprocessing and tract-based analysis of the DTI data was implemented using the FMRIB Software Library [26], the DTI ToolKit (http://www.nitrc.org/projects/dtitk/) and Tract-based spatial statistics [27]. Statistics were computed for fractional anisotropy and mean diffusivity using 10000 permutations and the contrast of patients versus controls and controls versus patients were considered. Again the influence of selected neuropsychological test results now on white matter changes were computed using permutation tests with 10000 permutations. Data were corrected for multiple comparison using threshold-free cluster enhancement and considered at a p-value of 0.05 [28]. Additionally, a region of interest analysis was performed using the IIT Human brain atlas template bundles as regions of interest and a 7 x 2 x 2 ANOVA (region x group x hemisphere) respectively a t-test.

## Results

### Patient characteristics

20 female patients with well-defined PBC (median age 55.5 [49.0–64.3] years) as well as 20 healthy age-matched female volunteers (median age 56.5 [44.3–65.0] years) were included. All PBC patients had detectable levels of PBC specific antibodies and received ursodeoxycholic acid. For detailed information about the patient cohort see Table 1.

**Table 1:** Patient characteristics.

Total number of patients	20 (100%)
**Additional symptoms**	
Sicca-Syndrome	8 (40%)
Arthralgia	3 (15%)
Pruritus	6 (30%)
**PBC specific antibodies**	
AMA	16 (80%)
gp210	1 (5%)
Sp-100	1 (5%)
AMA/Sp-100	1 (5%)
AMA/gp210/Sp-100	1 (5%)
**Liver cirrhosis**	1 (5%)
**UDCA treatment**	20 (100%)
**Liver tests at study entry (reference range)**	**Median value (Q1-Q3)**
Liver elastography*	6.4 kPa (5.1–6.9)
ALP (35–104)	120.5 U/l (58.8–167.5)
ALT (10–35)	25.0 U/l (19.3–43.8)
Total Bilirubin (<1.2)	0.5 mg/dl (0.4–0.7)
IgM (0.4–2.3)	1.9 g/l (1.3–2.3)
INR (<1.3)	1.0 (0.9–1.0)

20 patients with primary biliary cholangitis (median age 55.5 [49.0–64.3] years) were included.

* Liver elastography was available in only 17/20 patients.

ALP, alkaline phosphatase; ALT, alanine aminotransferase; AMA, antimitochondrial antibody; gp210, nuclear pore glycoprotein-210; IgM, Immunoglobulin M; INR, international normalized ratio; Sp-100, soluble acidic protein-100; UDCA, ursodeoxycholic acid.

### Assessment of fatigue und psychological symptoms

**Fig 1:**
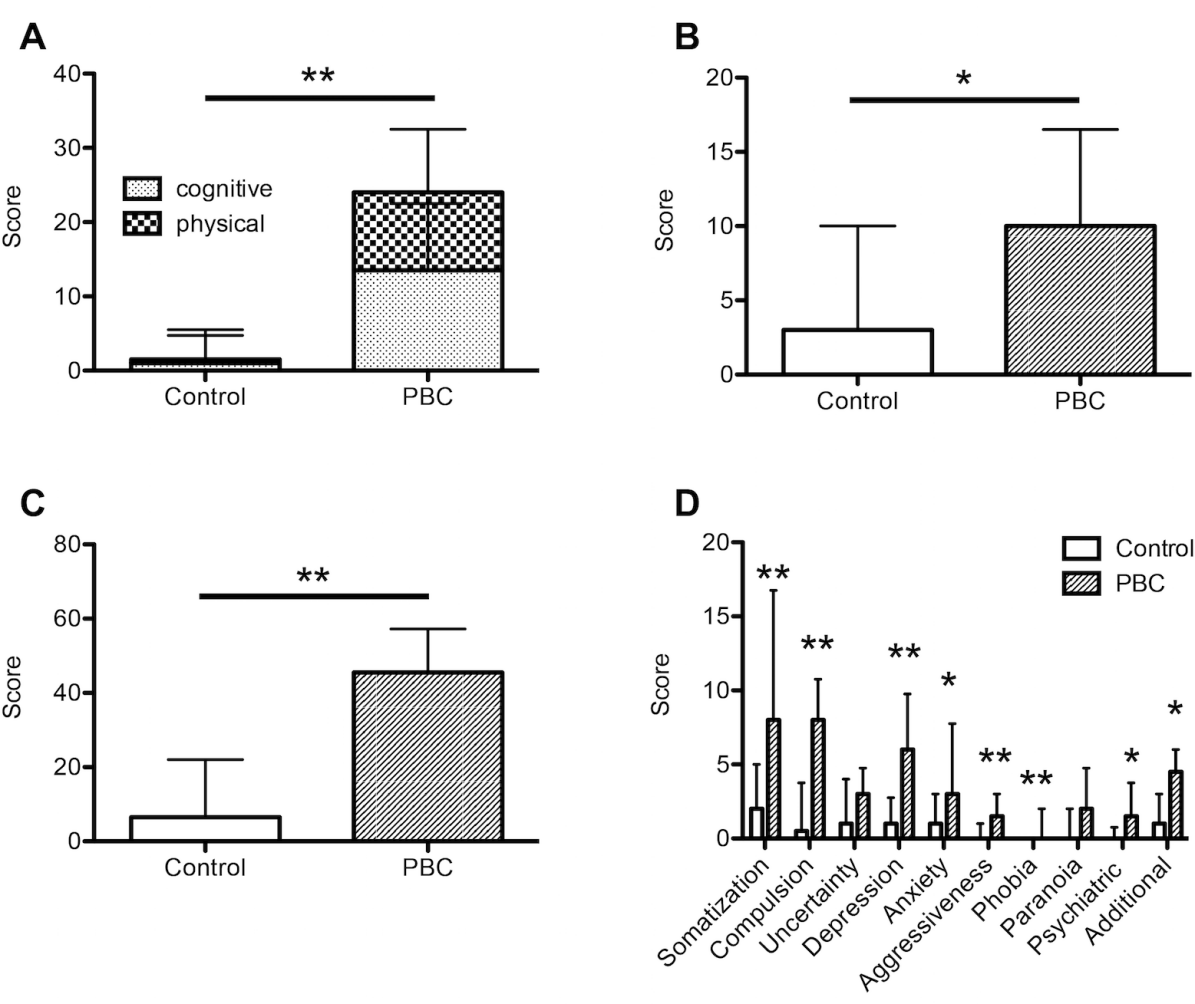
Assessment of fatigue and psychological symptoms. 20 patients with PBC and 20 age- and sex-matched controls were assessed with respect to fatigue and psychological symptoms. Compared to healthy controls, patients with PBC had significantly higher levels of fatigue (WEIMUS, A), depressive (BDI, B) as well as psychological symptoms (SCL-90-R). C presents the total SCL-90-R score, while D shows results from SCL-90-R subgroup analysis. BDI, Beck Depression Inventory; PBC, primary biliary cholangitis; SCL-90-R, Symptom Check List of Derogatis; WEIMUS, Würzburg depletion Inventory for multiple sclerosis. *, p<0.05; **, p<0.01 (Wilcoxon matched pairs test).

Consistent with the patient selection, fatigue was found to be significantly severer in the PBC group as compared to healthy controls, both with respect to cognitive and physical as well as total fatigue (median total WEIMUS score PBC vs. Control: 26.0 [10.3–41.8] vs. 1.5 [0.0–8.5]; p<0.01). In addition, patients with PBC were significantly more affected by depressive (BDI) and psychological symptoms (SCL-90-R) (Fig 1A–1C, Table 2). The subanalysis of the SCL-90-R score demonstrated that except for paranoid symptoms and uncertainty this difference was significant in every subscore, including somatization, compulsive behavior, depression, anxiety, aggressiveness, phobia as well as general psychiatric and additional symptoms (Fig 1D).

**Table 2:** Neuropsychological assessment.

Feature	Control-group Median value (Q1-Q3)	PBC-group Median value (Q1-Q3)	p-value
**Fatigue/psychological assessment**			
WEIMUS (total)	1.5 (0.0–8.5)	26.0 (10.3–41.8)	**<0.01**
BDI	3.0 (0.0–10.0)	10.0 (4.3–16.5)	**0.01**
SCL-90-R (total)	6.5 (3.0–22.0)	45.5 (21.0–57.3)	**<0.01**
**Cognitive assessment**			
Digit span	10.0 (8.0–11.0)	9.0 (8.0–11.0)	0.88
Digit-Ordering A	5.0 (4.0–7.0)	6.0 (4.3–6.0)	0.58
Digit-Ordering B	6.0 (4.0–6.8)	6.5 (5.3–7.8)	0.08
Lexical verbal fluency	24.0 (17.5–28.8)	19.0 (17.0–22.5)	**<0.01**
Semantic verbal fluency	39.5 (35.3–45.8)	33.5 (28.0–38.8)	**<0.01**
Trailmaking A	28.5 s (21.8–38.3)	30.0 s (26.3–35.0)	0.53
Trailmaking B	73.5 s (60.0–89.3)	81.5 s (63.5–96.5)	0.47
**Computerized test battery of attention performance (TAP)**			
Alertness I w/o signal	257.5 ms (236.5–296.0)	278.0 ms (237.8–324.8)	0.30
Alertness I with signal	250.5 ms (226.0–296.3)	264.5 ms (234.5–309.0)	0.16
Divided attention (auditive)	583.0 ms (529.8–663.3)	597.5 ms (533.5–634.3)	0.54
Divided attention (visual)	826.0 ms (790.5–953.5)	930.5 ms (848.5–992.0)	0.33
Vigilance (1^st^ half)	838.0 ms (781.3–944.8)	848.0 ms (758.3–927.8)	0.99
Vigilance (2^nd^ half)	861.0 ms (791.0–998.5)	919.0 ms (784.5–1052.0)	0.38
Vigilance (total)	848.0 ms (785.5–968.8)	881.5 ms (797.5–955.0)	0.77
Alertness II w/o signal	260.5 ms (241.8–290.0)	259.0 ms (240.8–299.8)	0.60
Alertness II with signal	243.0 ms (224.3–268.0)	244.5 ms (235.3–281.8)	0.37

Compared to healthy controls, patients with PBC (primary biliary cholangitis) had significantly higher scores with respect to fatigue (WEIMUS, Würzburg depletion Inventory for multiple sclerosis) and psychological symptoms (BDI, Beck depression inventory; SCL-90-R, Symptom check list of Derogatis). While the most subtests of the cognitive assessment revealed no differences between both groups, PBC patients had a significant impairment of both lexical and semantic verbal fluency. In respect to attention performance, no difference was found between patients and controls using the computerized test battery of attention performance (TAP, values given: reaction time). Statistical analysis: Wilcoxon matched pairs test.

### Cognitive assessment

**Fig 2:**
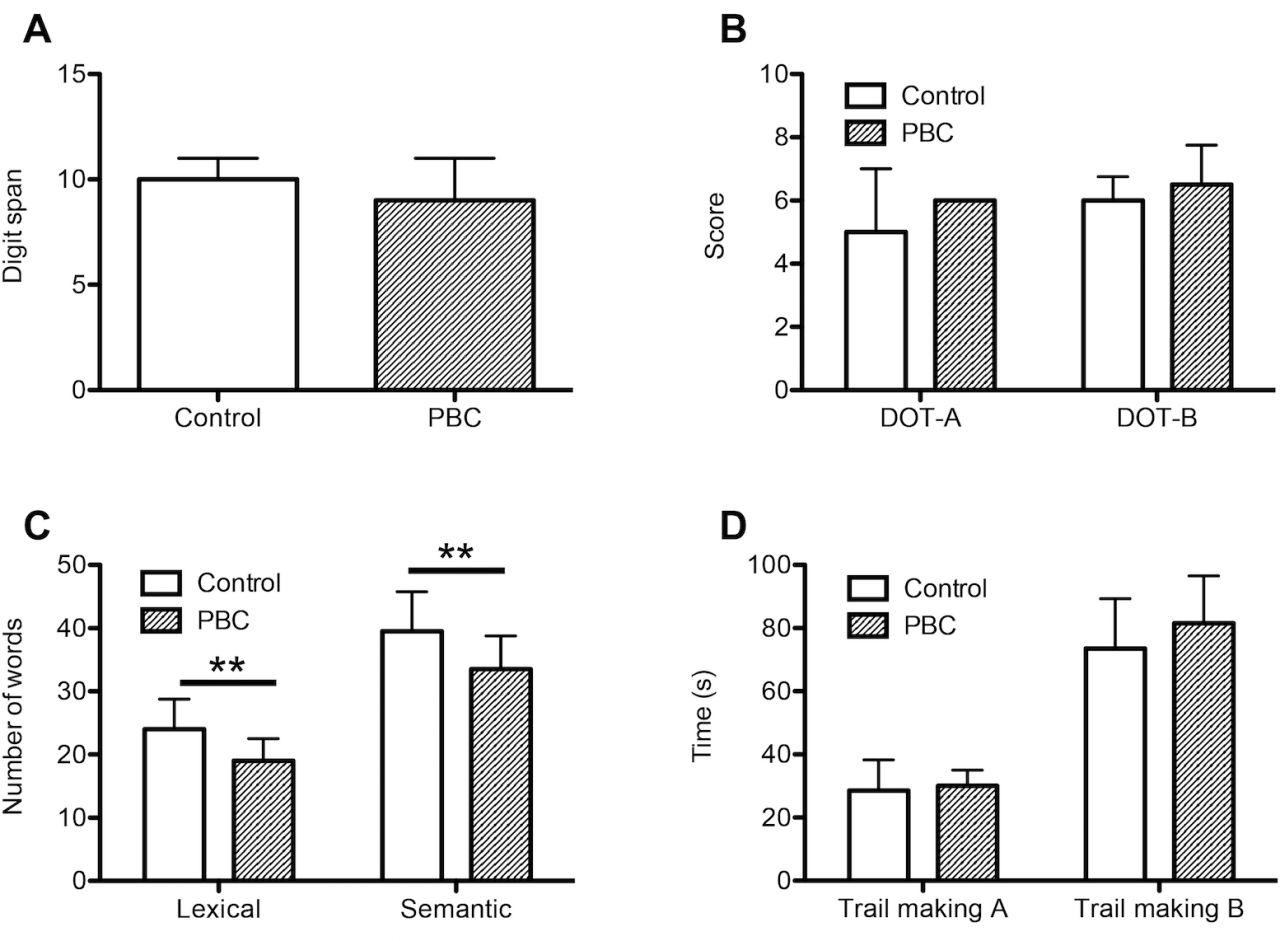
Cognitive assessment. 20 patients with PBC and 20 age- and sex-matched controls underwent a cognitive assessment. Compared to healthy controls, patients with PBC had a significant impairment of lexical and semantic verbal fluency (C), while results from digit span (A), digit ordering A and B (B) and trail making A and B (D) tests were similar, indicating no differences with respect to working memory and cognitive flexibility between both groups. DOT-A, digit ordering A; DOT-B, digit ordering B; PBC, primary biliary cholangitis; **, p<0.01 (Wilcoxon matched pairs test).

The cognitive assessment revealed no differences with respect to working memory (digit span, digit ordering A/B) and cognitive flexibility (trailmaking A/B) between PBC patients and controls. Results from verbal fluency assessment, however, demonstrated a significant impairment of PBC patients´ lexical (number of words named PBC vs. Control: 19.0 [17.0–22.5] vs. 24.0 [17.5–28.8]; p<0.01) and semantic verbal fluency (number of words named PBC vs. Control: 33.5 [28.0–38.8] vs. 39.5 [35.3–45.8]; p<0.01) (Fig 2, Table 2).

### Computerized test battery of attention performance

**Fig 3:**
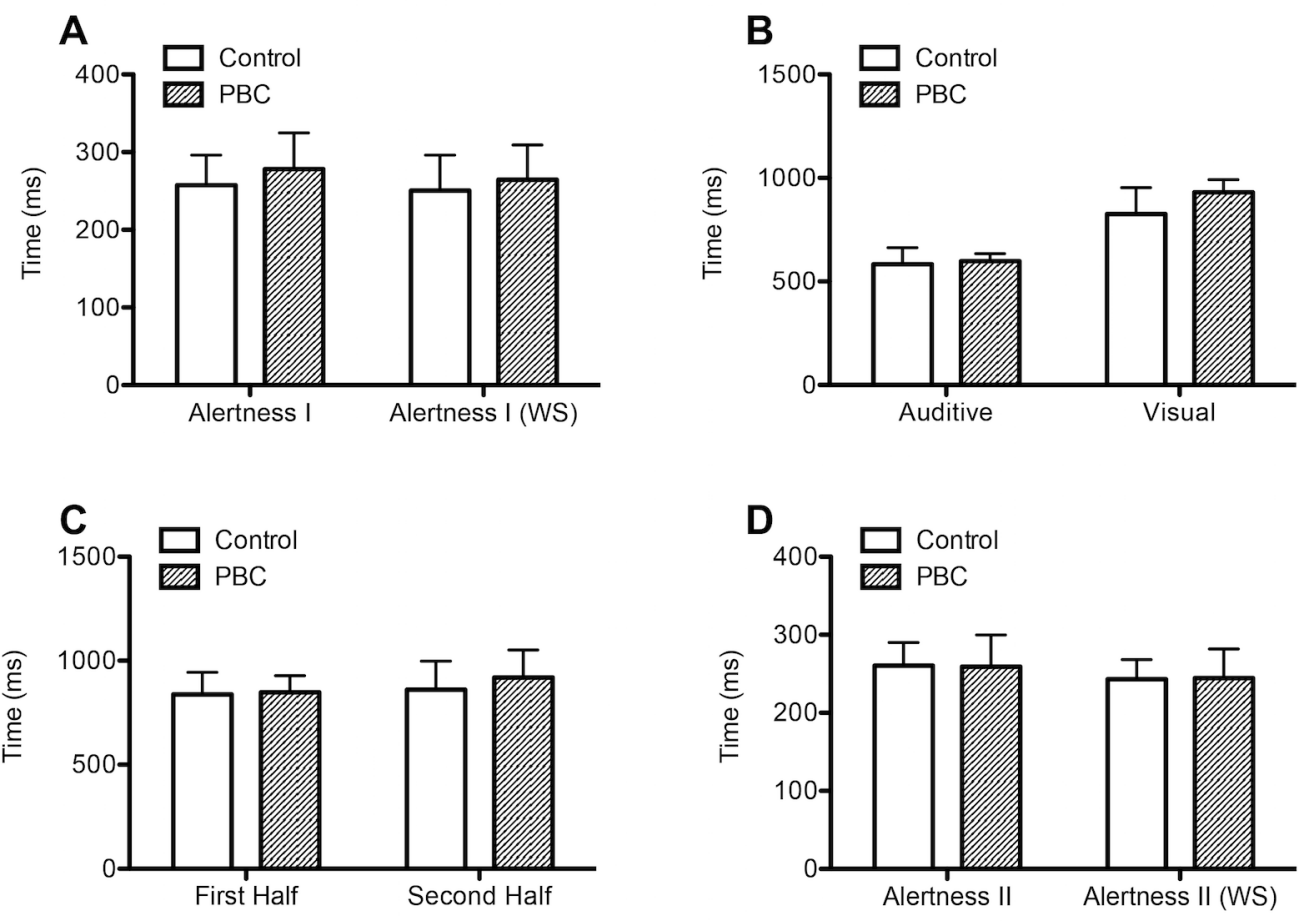
Computerized test battery of attention performance. 20 patients with PBC and 20 age- and sex-matched controls underwent a computerized test battery of attention performance (TAP) in order of the figure appearance. No differences of reaction time were observed between both groups within all subtests (Wilcoxon matched pairs test). Alertness I without and with warning signal (WS) (A), divided attention auditive/visual (B), 30 minutes vigilance test first half/second half (C), alertness II without and with warning signal (WS) (D). PBC, primary biliary cholangitis.

Using the computerized test battery of attention performance (TAP) no differences were detected with respect to the study participants´ reaction time in the subtest alertness (alertness I, with/without warning signal), divided attention (auditive/visual) or vigilance (Fig 3A–3C, Table 2).

In addition, no differences of test results were observed when repeating the subtest alertness at the end of the TAP (alertness II, Fig 3D, Table 2), nor did the PBC patients´ reaction time deteriorate more between the first and second half during the subtest vigilance (F(1, 38) = 2.52, p = 0.12, two-way ANOVA).

### Voxel-based morphometry

**Fig 4:**
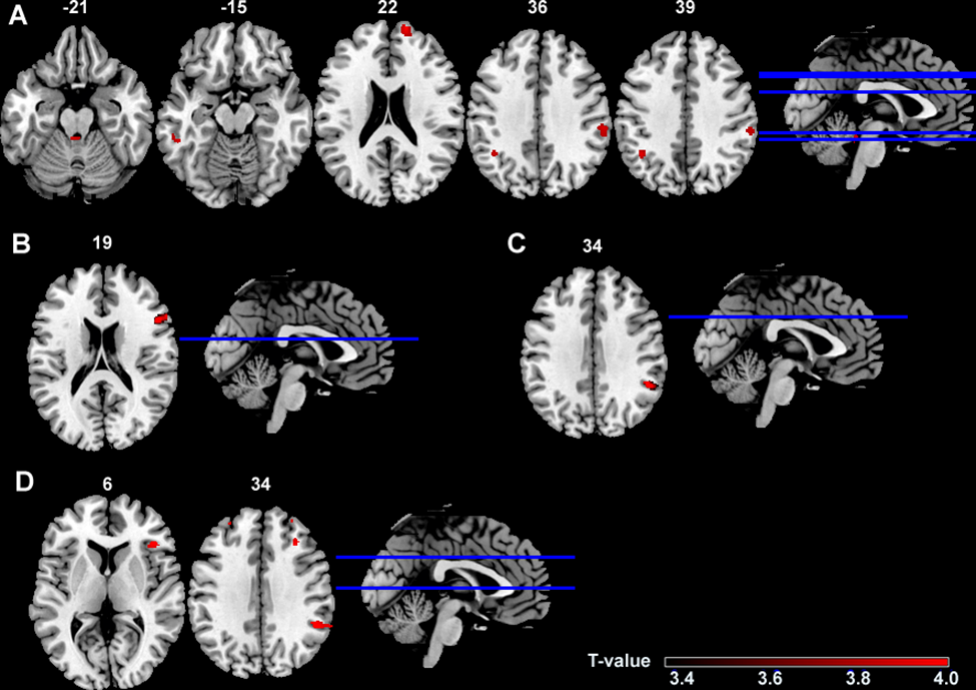
Voxel-based morphometry. Compared to healthy controls, patients with primary biliary cholangitis showed decreased grey matter volume in the superior frontal, inferior parietal, inferior temporal and supramarginal gyrus and brainstem (A). In the overall cohort, a worse performance in the semantic verbal fluency (percentile rank) (B) correlated with decreased grey matter volume in the right inferior frontal gyrus. A better reaction time in the subtest alertness I (with WS) (C) correlated to a decreased grey matter volume whereas a worse (higher) reaction time in the subtest alertness II (with WS) (D) correlated to decreased grey matter in the supramarginal and right frontal gyrus. Note, that data shown are uncorrected (p<0.001) and of exploratory nature. Plotted on the ch2bet brain template in MRIcron. The color-scale shows the range of T-values. MNI, Montreal Neurological Institute (MNI) z coordinates; WS, warning signal.

Voxel-based morphometry (VBM) revealed no significant changes in the grey matter volume of PBC patients with fatigue compared to healthy controls if corrected for multiple comparisons (false discovery rate corrected p-value of 0.05). To detect grey matter changes that may become significant at a larger sample size an additional exploratory analysis at a more lenient threshold (p<0.001, cluster threshold 50 voxel) was performed. This analysis showed a reduction of the PBC patients´ grey matter volume in the right superior frontal gyrus, the left inferior temporal and inferior parietal gyrus as well as in the right supramarginal gyrus and in the brainstem (Fig 4A, Table 3A).

**Table 3:** Voxel-based morphometry.

		Brain region	Hemis-phere	MNI Coordinates			Size	T-value
				x	y	z		
**Decrease of grey matter volume in PBC compared to controls (A)**		Frontal Sup	R	16	63	22	226	4.90
		Temporal Inf	L	-51	-39	-15	52	4.31
		Parietal Inf	L	-40	-52	39	91	4.28
		Supramarginal	R	60	-28	36	160	4.16
		Brainstem	L	-3	-38	-21	56	4.04
**Correlation of decreased grey matter volume within the overall cohort with…**	**Reduced semantic verbal fluency (B)**	Frontal Inf Tri	R	51	18	20	262	4.31
	**Increased alertness (alertness I with WS) (C)**	Supramarginal	R	51	-46	34	142	4.52
	**Decreased alertness (alertness II with WS) (D)**	Supramarginal	R	50	-45	34	197	4.47
		Frontal Inf Tri	R	42	28	6	62	4.25
		Frontal Mid	R	32	27	34	91	3.93

VBM showed decreased grey matter volumes of distinct brain regions in patients with primary biliary cholangitis (PBC) compared to the control group (A). Within the whole study population, a worse performance in the semantic verbal fluency (percentile rank) (B) correlated with decreased grey matter volume in the right inferior frontal gyrus. A better reaction time in the subtest alertness I (with WS) (C) correlated to a decreased grey matter volume whereas a worse (higher) reaction time in the subtest alertness II (with WS) (D) correlated to decreased grey matter. Note, that data shown are uncorrected (p<0.001) and of exploratory nature. MNI (Montreal Neurologic Institute) coordinates. Size in number of voxels, cluster threshold 50 voxel. Inf, inferior; L, left; Mid, middle; R, right; Sup, superior; Tri, triangularis; WS, warning signal.

Furthermore, data from VBM analysis were correlated to results from selected neuropsychological tests in the overall cohort. No significant results were detected for the correlation between the study participants´ grey matter volume and their semantic verbal fluency (percentile rank), alertness (reaction time alertness I and II with warning signal, TAP) or vigilance (total reaction time vigilance, TAP) (false discovery rate corrected p-value of 0.05). Additionally, an exploratory analysis at a threshold of p<0.001 (cluster threshold 50 voxel) was performed for correlation analysis, as well. Results from the uncorrected correlation analysis are provided in Fig 4B–4D and Table 3B–3D.

### Diffusion tensor imaging

The DTI analysis revealed no differences of fractional anisotropy and mean diffusivity between PBC patients and healthy controls. Furthermore, within the whole cohort fractional anisotropy and mean diffusivity did not correlate to the study participants´ fatigue (total WEIMUS score), semantic verbal fluency (percentile rank), alertness (reaction time alertness I and II with warning signal, TAP) or vigilance (total reaction time vigilance, TAP). Additionally, the region of interest analysis of the DTI data did not reveal significant results.

## Discussion

Previous studies have pointed out the possibility of CNS alterations in patients with PBC as a pathogenetic mechanism for fatigue, but data on structural brain abnormalities are inconsistent [7–11,14]. By the combined use of clinical scales, an objective assessment of cognitive and attention performance as well as structural brain imaging with T1-weighted cMRI and DTI we sought to shed further light on this issue. In spite of marked fatigue and psychological symptoms, PBC patients in our study had neither major cognitive or attention deficits, nor significant structural brain abnormalities.

This appears to be in contrast to a number of previous studies. For example, Forton et al. found differences of the globus pallidus magnetization transfer ratio in 14 pre-cirrhotic patients compared to healthy controls, which was correlated to fatigue. An accumulation of manganese, which is normally excreted via the biliary route, was suggested underlying both, fatigue and MRI-changes [10]. On the other hand Hollingsworth et al. found altered T2 values in the white matter and globus pallidus in early-stage PBC patients compared to healthy controls, while T1 values of the globus pallidus were similar between both groups, arguing against altered globus pallidus manganese levels in pre-cirrhotic PBC [11]. Finally, by using T2-weighted cMRI Newton et al. described predominantly white matter lesions in 11 PBC patients and found a correlation between the total lesion load and the patients´ cognitive decline [9].

In the present study we used DTI, thus enabling us to reveal early structural abnormalities of white matter. While PBC patients in the current sample showed the typical signs of fatigue, our data argue against white matter alterations as a pathogenetic factor for fatigue in PBC. In particular, we found neither a decrement of white matter in the patients ‘group nor a correlation between fatigue scores or results from vigilance and attention tests with DTI measures employing both, a whole brain and a region of interest approach. At first glance, this is in contrast to a recently published pilot study by Grover et al., who described an increased mean diffusivity in the thalamus of 13 early-stage PBC patients [14]. However, compatible to our results, no differences were found in other parts of the CNS besides the thalamus, nor did fractional anisotropy differ between patients and controls, indicating that structural abnormalities of white matter play a minor role in the pathogenesis of fatigue in PBC.

In addition we also employed VBM on T1-weighted high-resolution images to detect changes in grey matter volume, which have been shown to be present in conditions associated with fatigue other than PBC before. For instance, in chronic fatigue syndrome, a rather ill-defined condition, both, increases and reductions of grey matter volume have been reported [29]. While in our cohort no significant grey matter alterations between PBC patients and healthy controls were detected at a very strict statistical threshold, an analysis with a more lenient threshold suggested reduced grey matter volume in the right superior frontal gyrus, the left inferior temporal and inferior parietal gyrus as well as in the right supramarginal gyrus and in the brainstem. Thus, slight alterations of grey matter appear to be present in PBC and may in general be related to the cognition and attention. These data, however, need to be replicated in an appropriately large cohort.

A major finding of our study is the dissociation of subjective fatigue as reflected by the WEIMUS score and the preserved performance in the cognitive tasks. This seems to contrast with previous studies that established the idea of a cognitive impairment in PBC patients with fatigue [4,8,9,15]. Most of these studies relied on the patients’ self-reports to assess cognitive impairment, thus limiting validity of the data. The only study employing formal cognitive testing reported significant impairment of PBC patients in all cognitive function tests but most pronounced for verbal fluency [9]. Interestingly, PBC patients performed significantly worse than controls for verbal fluency also in the present study.

Previously, fatigue in PBC has been associated with an increased daytime drowsiness [30], suggesting an impairment of attention in PBC as well. Using a computerized assessment to measure attention performance, our study for the first time evaluated, whether attention deficits are present in PBC patients with severe fatigue. Interestingly, no attention impairment was detected in our patient cohort, nor did PBC patients show a more severe deterioration of attention over time than healthy controls. This may have positive implications for a variety of aspects in daily life, e.g. with respect to driving ability, that has been found to be reduced in conditions other than fatigue, such as minimal hepatic encephalopathy [31].

We selected PBC patients specifically for a high degree of fatigue, which prevented us from a reasonable correlation of the fatigue severity with other test results, e.g. cMRI data, within the patient group. This approach was chosen to enhance the probability of detecting structural brain abnormalities, if present, making it unlikely that structural brain alterations associated with fatigue were underestimated in our study. A potential limitation of the current study is the use of the WEIMUS scale, developed for assessment of fatigue in multiple sclerosis. We chose this scale, as it is available in a validated form in German, which is an advantage over PBC-40 and the Fatigue Impact Scale (FIS) addressing fatigue in PBC patients [15,16]. As fatigue symptoms cut across diagnostic boundaries and, moreover, the WEIMUS differentiates between physical and cognitive fatigue, we considered this instrument to resemble the domains “fatigue” and “cognitive function” of the PBC-40 best and therefore to be most appropriate for the present purpose.

To summarize, the relevance of fatigue and the high burden of associated psychological symptoms was confirmed in a cohort of patients with PBC. Except for a modest impairment of verbal fluency, patients with PBC were not impaired with regard to cognitive and attention performance. Moreover, PBC was not associated with major structural brain abnormalities of either grey or white matter. We take these findings to suggest functional rather than structural changes underlying fatigue in PBC. These functional alterations would be best assessed in a heterogeneous cohort of PBC patients with and without fatigue using measures such as event-related potentials or resting state functional MRI [32,33].

## Supporting information

S1 Appendix Materials and methods. (DOCX)

## References

[1] European Association for the Study of the L (2009) EASL Clinical Practice Guidelines: management of cholestatic liver diseases. J Hepatol 51: 237–267. doi: 10.1016/j.jhep.2009.04.009 19501929 10.1016/j.jhep.2009.04.009

[2] Huet PM, Deslauriers J, Tran A, Faucher C, Charbonneau J (2000) Impact of fatigue on the quality of life of patients with primary biliary cirrhosis. Am J Gastroenterol 95: 760–767. doi: 10.1111/j.1572-0241.2000.01857.x 10710071 10.1111/j.1572-0241.2000.01857.x

[3] Jones DE, Al-Rifai A, Frith J, Patanwala I, Newton JL (2010) The independent effects of fatigue and UDCA therapy on mortality in primary biliary cirrhosis: results of a 9 year follow-up. J Hepatol 53: 911–917. doi: 10.1016/j.jhep.2010.05.026 20800924 10.1016/j.jhep.2010.05.026

[4] Jopson L, Jones DE (2015) Fatigue in Primary Biliary Cirrhosis: Prevalence, Pathogenesis and Management. Dig Dis 33 Suppl 2: 109–114. 26641884 10.1159/000440757

[5] Carbone M, Bufton S, Monaco A, Griffiths L, Jones DE, Neuberger JM (2013) The effect of liver transplantation on fatigue in patients with primary biliary cirrhosis: a prospective study. J Hepatol 59: 490–494. doi: 10.1016/j.jhep.2013.04.017 23628322 10.1016/j.jhep.2013.04.017

[6] Zenouzi R, Weiler-Normann C, Lohse AW (2013) Is fatigue in primary biliary cirrhosis cured by transplantation? J Hepatol 59: 418–419. doi: 10.1016/j.jhep.2013.05.037 23742911 10.1016/j.jhep.2013.05.037

[7] Burak KW, Le T, Swain MG (2001) Increased midbrain 5-HT1A receptor number and responsiveness in cholestatic rats. Brain Res 892: 376–379. 11172786 10.1016/s0006-8993(00)03058-4

[8] Newton JL, Bhala N, Burt J, Jones DE (2006) Characterisation of the associations and impact of symptoms in primary biliary cirrhosis using a disease specific quality of life measure. J Hepatol 44: 776–783. doi: 10.1016/j.jhep.2005.12.012 16487619 10.1016/j.jhep.2005.12.012

[9] Newton JL, Hollingsworth KG, Taylor R, El-Sharkawy AM, Khan ZU, Pearce R, et al (2008) Cognitive impairment in primary biliary cirrhosis: symptom impact and potential etiology. Hepatology 48: 541–549. doi: 10.1002/hep.22371 18563843 10.1002/hep.22371

[10] Forton DM, Patel N, Prince M, Oatridge A, Hamilton G, Goldblatt J, et al (2004) Fatigue and primary biliary cirrhosis: association of globus pallidus magnetisation transfer ratio measurements with fatigue severity and blood manganese levels. Gut 53: 587–592. doi: 10.1136/gut.2003.016766 15016756 10.1136/gut.2003.016766 PMC1774014

[11] Hollingsworth KG, Jones DE, Aribisala BS, Thelwall PE, Taylor R, Newton JL, et al (2009) Globus pallidus magnetization transfer ratio, T(1) and T(2) in primary biliary cirrhosis: relationship with disease stage and age. J Magn Reson Imaging 29: 780–784. doi: 10.1002/jmri.21555 19306399 10.1002/jmri.21555

[12] Soares JM, Marques P, Alves V, Sousa N (2013) A hitchhiker's guide to diffusion tensor imaging. Front Neurosci 7: 31 doi: 10.3389/fnins.2013.00031 23486659 10.3389/fnins.2013.00031 PMC3594764

[13] Wilting J, Rolfsnes HO, Zimmermann H, Behrens M, Fleischer V, Zipp F, et al (2016) Structural correlates for fatigue in early relapsing remitting multiple sclerosis. Eur Radiol 26: 515–523. doi: 10.1007/s00330-015-3857-2 26026721 10.1007/s00330-015-3857-2

[14] Grover VP, Southern L, Dyson JK, Kim JU, Crossey MM, Wylezinska-Arridge M, et al (2016) Early primary biliary cholangitis is characterised by brain abnormalities on cerebral magnetic resonance imaging. Aliment Pharmacol Ther 44: 936–945. doi: 10.1111/apt.13797 27604637 10.1111/apt.13797 PMC5082539

[15] Jacoby A, Rannard A, Buck D, Bhala N, Newton JL, James OF, et al (2005) Development, validation, and evaluation of the PBC-40, a disease specific health related quality of life measure for primary biliary cirrhosis. Gut 54: 1622–1629. doi: 10.1136/gut.2005.065862 15961522 10.1136/gut.2005.065862 PMC1774759

[16] Prince MI, James OF, Holland NP, Jones DE (2000) Validation of a fatigue impact score in primary biliary cirrhosis: towards a standard for clinical and trial use. J Hepatol 32: 368–373. 10735604 10.1016/s0168-8278(00)80385-2

[17] Flachenecker P, Muller G, Konig H, Meissner H, Toyka KV, Rieckmann P (2006) ["Fatigue" in multiple sclerosis. Development and and validation of the "Wurzburger Fatigue Inventory for MS"]. Nervenarzt 77: 165–166, 168–170, 172–164. doi: 10.1007/s00115-005-1990-x 16160812 10.1007/s00115-005-1990-x

[18] Beck AT, Ward CH, Mendelson M, Mock J, Erbaugh J (1961) An inventory for measuring depression. Arch Gen Psychiatry 4: 561–571. 13688369 10.1001/archpsyc.1961.01710120031004

[19] Derogatis LR, Rickels K, Rock AF (1976) The SCL-90 and the MMPI: a step in the validation of a new self-report scale. Br J Psychiatry 128: 280–289. 1252693 10.1192/bjp.128.3.280

[20] Hoppe CD, Muller UD, Werheid KD, Thone AD, von Cramon YD (2000) Digit Ordering Test: clinical, psychometric, and experimental evaluation of a verbal working memory test. Clin Neuropsychol 14: 38–55. doi: 10.1076/1385-4046(200002)14:1;1-8;FT038 10855058 10.1076/1385-4046(200002)14:1;1-8;FT038

[21] Arbuthnott K, Frank J (2000) Trail making test, part B as a measure of executive control: validation using a set-switching paradigm. J Clin Exp Neuropsychol 22: 518–528. doi: 10.1076/1380-3395(200008)22:4;1-0;FT518 10923061 10.1076/1380-3395(200008)22:4;1-0;FT518

[22] Corrigan JD, Hinkeldey NS (1987) Relationships between parts A and B of the Trail Making Test. J Clin Psychol 43: 402–409. 3611374 10.1002/1097-4679(198707)43:4<402::aid-jclp2270430411>3.0.co;2-e

[23] Gaudino EA, Geisler MW, Squires NK (1995) Construct validity in the Trail Making Test: what makes Part B harder? J Clin Exp Neuropsychol 17: 529–535. doi: 10.1080/01688639508405143 7593473 10.1080/01688639508405143

[24] Aschenbrenner S, O T, K L (2000) Regensburger Wortflüssigkeitstest. Lisse: Swets und Zeitlinger Verlag.

[25] Genovese CR, Lazar NA, Nichols T (2002) Thresholding of statistical maps in functional neuroimaging using the false discovery rate. Neuroimage 15: 870–878. doi: 10.1006/nimg.2001.1037 11906227 10.1006/nimg.2001.1037

[26] Jenkinson M, Beckmann CF, Behrens TE, Woolrich MW, Smith SM (2012) Fsl. Neuroimage 62: 782–790. doi: 10.1016/j.neuroimage.2011.09.015 21979382 10.1016/j.neuroimage.2011.09.015

[27] Smith SM, Jenkinson M, Johansen-Berg H, Rueckert D, Nichols TE, Mackay CE, et al (2006) Tract-based spatial statistics: voxelwise analysis of multi-subject diffusion data. Neuroimage 31: 1487–1505. doi: 10.1016/j.neuroimage.2006.02.024 16624579 10.1016/j.neuroimage.2006.02.024

[28] Smith SM, Nichols TE (2009) Threshold-free cluster enhancement: addressing problems of smoothing, threshold dependence and localisation in cluster inference. Neuroimage 44: 83–98. doi: 10.1016/j.neuroimage.2008.03.061 18501637 10.1016/j.neuroimage.2008.03.061

[29] Tang LW, Zheng H, Chen L, Zhou SY, Huang WJ, Li Y, et al (2015) Gray matter volumes in patients with chronic fatigue syndrome. Evid Based Complement Alternat Med 2015: 380615 doi: 10.1155/2015/380615 25792998 10.1155/2015/380615 PMC4352504

[30] Newton JL, Gibson GJ, Tomlinson M, Wilton K, Jones D (2006) Fatigue in primary biliary cirrhosis is associated with excessive daytime somnolence. Hepatology 44: 91–98. doi: 10.1002/hep.21230 16800007 10.1002/hep.21230

[31] Bajaj JS, Saeian K, Schubert CM, Hafeezullah M, Franco J, Varma RR, et al (2009) Minimal hepatic encephalopathy is associated with motor vehicle crashes: the reality beyond the driving test. Hepatology 50: 1175–1183. doi: 10.1002/hep.23128 19670416 10.1002/hep.23128 PMC2757520

[32] Boissoneault J, Letzen J, Lai S, O'Shea A, Craggs J, Robinson M, et al (2015) Abnormal resting state functional connectivity in patients with chronic fatigue syndrome: An arterial spin-labeling fMRI study. Magn Reson Imaging. 10.1016/j.mri.2015.12.008 PMC4801728 26708036

[33] Nagels G, D'Hooghe M B, Vleugels L, Kos D, Despontin M, De Deyn PP (2007) P300 and treatment effect of modafinil on fatigue in multiple sclerosis. J Clin Neurosci 14: 33–40. doi: 10.1016/j.jocn.2005.10.008 17138067 10.1016/j.jocn.2005.10.008

