# Supplementary material for: Patients with primary biliary cholangitis and fatigue present with depressive symptoms and selected cognitive deficits, but with normal attention performance and brain structure
Source: PLoS One. 2018 Jan 10;13(1):e0190005. doi: 10.1371/journal.pone.0190005 (PMC5761833; doi:10.1371/journal.pone.0190005)
Supplement: S1 Appendix — (DOCX) [file pone.0190005.s001.docx]

**Patients with primary biliary cholangitis and fatigue present with depressive symptoms and selected cognitive deficits, but with normal attention performance and brain structure**

**Short title: Assessment of structural brain changes in PBC patients with fatigue**

Roman Zenouzi^1¶*^, Janina von der Gablentz^2¶^, Marcus Heldmann^2^, Martin Göttlich^2^, Christina Weiler-Normann^1^, Marcial Sebode^1^, Hanno Ehlken^1,3^, Johannes Hartl^1^, Anja Fellbrich^2^, Susanne Siemonsen^4^, Christoph Schramm^1^, Thomas F. Münte^2&^, Ansgar W. Lohse^1&^

^1^ 1^st^ Department of Medicine, University Medical Center Hamburg-Eppendorf, Hamburg, Germany.

^2^ Department of Neurology, University of Lübeck, Lübeck, Germany.

^3^ Department of Interdisciplinary Endoscopy, University Medical Center Hamburg-Eppendorf, Hamburg, Germany.

^4^ Department of Diagnostic and Interventional Neuroradiology, University Medical Center Hamburg-Eppendorf, Hamburg, Germany.

^¶^ authors share co-first authorship

^&^ senior authors contributed equally to this work

^*^ corresponding author (r.zenouzi@uke.de)

**Supporting information – S1 appendix**

Materials and Methods p. 2

Experimental procedure p. 2

Voxel-based morphometry analysis p. 4

Diffusion tensor imaging analysis p. 6

References p. 7

**Materials and Methods**

**Experimental procedure**

For all subjects, the experimental procedure included clinical scales to assess fatigue and psychological symptoms (i), a cognitive assessment (ii), a computerized test battery of attention performance (iii) as well as the cerebral magnetic resonance imaging (cMRI) measurement (iv).

(i) For fatigue assessment the Würzburg depletion Inventory for multiple sclerosis (WEIMUS, German, Würzburger Erschöpfungsinventar für Multiple Sklerose), which is a validated instrument to measure fatigue in patients with multiple sclerosis and differentiates between physical and cognitive fatigue, was used [1]. Psychological symptoms potentially associated to fatigue were assessed with the Beck Depression Inventory (BDI), which is a standardized self-report questionnaire with 21 items regarding depressive symptoms [2], and the Symptom check list of Derogatis (SCL-90-R) [3]. The latter enquires 90 items, which reflect psychological symptoms of different subgroups (e.g. anxiety, somatization) that may be associated with fatigue.

(ii) The cognitive assessment addressed working memory, cognitive flexibility and verbal fluency. To examine working memory participants had to repeat numbers with a maximum span of 10 digits (digit span) and order numbers with a maximum span of 8 digits in ascending order (digit ordering test A, DOT-A) [4]. Afterwards the digit ordering test was repeated with one number mentioned twice, so the participant had to order the numbers again but also name the one number twice (DOT-B). The number of correct answers was registered for the analyses. To assess participants´ cognitive flexibility a trail making test consisting of two parts was used. In part A participants had to connect numbers in ascending order as fast as possible. In part B numbers had to be connected alternately to letters (e.g. 1-A-2-B). Mistakes occurring during the tests were pointed out to the participant by the test administrator, in order to be corrected during the test performance, which costs time, though. The time required for the tasks were recorded by the administrator [5-7]. Additionally, a standardized verbal fluency test (German, Regensburger Wortflüssigkeitstest) [8] was applied with a lexical and semantic subtest to assess the ability to solve problems. The participants had to name as many words as possible with the initial letter “S” (lexical) and from the category “animals” (semantic) within two minutes. The number of correct words was recorded for the analysis.

(iii) To measure different components of attention a computerized test battery of attention performance (TAP, PSYTEST, Herzogenrath, Germany) was used (subtests alertness, divided attention, vigilance and alertness in this sequence; duration: approximately 45 minutes). In every test the participant had to react to a stimulus as fast as possible by pressing a button. Reaction time was recorded. During the subtest alertness the participant had to react on a visual stimulus presented on a screen without or with an acoustic warning signal before the stimulus presentation in order to investigate the tonic and phasic alertness. The subtest alertness was tested once at the beginning (alertness I) and repeated at the end of the test battery (alertness II) to evaluate the effect of a long concentration period on test performance. Divided attention is often demanded in daily situations and was measured by the presentation of crosses on the screen occasionally forming a square that had to be detected by the participants (divided attention visual). Concurrently, an acoustic stimulus series was presented with alternating high and low tones with the target being a repetition of either the high or the low tone (divided attention auditive). Vigilance was determined by presentation of a beam which drifted vertically up and down with varying velocity over the display for a duration of 30 minutes. The participant was called to react every time the amplitude raised. The reaction time was measured separately for the first and second half of the test to detect deterioration over time.

(iv) Structural images were acquired on a 3 Tesla Siemens MAGNETOM Skyra scanner with a 20-Channel head coil. We recorded a T1-weighted 3D dataset with a MPRAGE sequence with a repetition time of 2400 ms, an echo time of 3.59 ms with 0.8 mm isotropic voxel and a field of view of 256 x 256. Diffusion-weighted sequences consisted of 50 axial slices with a 128 x 128 matrix, 2 mm slice thickness and 2 mm interslice gap. They were measured using a repetition time of 7200 ms, an echo time of 86 ms and an 80° flip angle.

**Voxel-based morphometry analysis**

Voxel-based morphometry (VBM) was carried out using Statistical Parametric Mapping 12b (SPM, http://www.fil.ion.ucl.ac.uk/spm) running with MATLAB 2013b (MATLAB and Statistics Toolbox Release 2013b, The MathWorks, Inc., Natick, Massachusetts, United States). The Diffeomorphic Anatomical Registration Through Exponentiated Lie Algebra (DARTEL) [9] toolbox was used for registration, because it is a robust and reliable approach for inter-subject registration of brain images for VBM analysis [10]. Initially, T1-weighted images of each subject were manually reoriented to the anterior commissure in alignment with the Montreal Neurologic Institute (MNI) coordinate system. The manually reoriented structural images were segmented into grey matter, white matter and cerebrospinal fluid volume probability maps using the new segment option of SPM which gives, additionally to the native space version, DARTEL imported versions of the tissues. For registration the DARTEL imported version of grey and white matter were used to iteratively generate a study-specific template and flow fields. The individual grey and white matter images were then normalized to MNI space using the normalize to MNI option of SPM with flow fields and the template generated in the previous step. Afterwards all images were smoothed with an 8 mm Full width at half maximum isotropic Gaussian kernel. Preprocessed data were visually inspected for registration errors.

For voxel-wise group statistics a two-sample t-test was generated with the patient and the control group. The age of all subjects was included as covariate because it is a potentially confounding factor. Gender was not included, because all participants were female. Additionally, we adjusted for each subject’s total intracranial volume (grey matter + white matter + cerebrospinal fluid) by entering the global values as covariate using ANCOVA correction. An absolute masking was implemented with a threshold of 0.15. The contrasts between healthy subjects and fatigue patients and vice versa were computed. The results were corrected for multiple testing using the false discovery rate correction [10] and considered at a corrected p-value of 0.05 with xjview (https://www.nitrc.org/projects/xjview/). Additionally, data were considered uncorrected at a p-value of 0.001 to get an impression of the grey matter differences.

The effects of the total WEIMUS score, reaction times from the vigilance and alertness tests (I and I with warning signal) and the percentile rank of the semantic verbal fluency task on the grey matter volume were computed using a multiple regression analysis with all subjects’ data, independent of their group and the different scores as covariates of interest. The results were again considered at a false discovery rate corrected p-value of 0.05 and uncorrected at a p-value of 0.001.

**Diffusion tensor imaging analysis**

Preprocessing and tract-based analysis was implemented using the FMRIB Software Library [11], the Diffusion Tensor Imaging ToolKit (http://www.nitrc.org/projects/dtitk/) and Tract-based spatial statistics [12]. For preprocessing eddy current related distortions and simple head motion were corrected to a reference volume using affine registration. Afterwards a brain mask was created with the middle b = 0 images and the tensor of each point in the mask was calculated. All fractional anisotropy (FA) maps were nonlinear registered to the IIT Human brain atlas template (http://www.nitrc.org/projects/iit2). By thinning the mean FA map with a threshold of 0.2 and keeping only the center of the white matter tracts an FA skeleton was obtained. Then, to avoid residual misalignment, for all subjects the voxels with the highest FA perpendicular to the tract were projected onto the skeleton. Voxel-wise comparison of white matter fibre tracts were then performed for FA, and mean diffusivity using Tract-based spatial statistics. Statistics were computed using the function randomize (http://fsl.fmrib.ox.ac.uk/fsl/fslwiki/Randomise/UserGuide) with 10000 random permutation tests. The contrasts patients versus controls and controls versus patients were performed. Additionally, the influence of the total WEIMUS score, the reaction time in the alertness (I and II with warning signal) and vigilance test and the percentile rank of the semantic verbal fluency task were calculated separately using the random permutation test again with 10000 permutations. Data were corrected for multiple comparison using threshold-free cluster enhancement [13] and considered at a corrected p-value of 0.05. Additionally, a region of interest analysis was performed using the IIT Human brain atlas template bundles as regions of interests. Using FSL each bundle was binarized with a threshold value of 0.15 and the mean value inside each region of interest was calculated for every subject. A 7 x 2 x 2 ANOVA (region x group x hemisphere) was performed with regions cingulate bundle, cingulate bundle 2, corticospinal tracts, inferior fronto-occipital fasciculus, superior and inferior longitudinal fasciculus and uncinated fasciculus. For three additional regions (forceps major, forceps minor, fornix) without hemisphere assignment of the IIT template bundles we separately calculated a t-test.

**References**

1. Flachenecker P, Muller G, Konig H, Meissner H, Toyka KV, Rieckmann P (2006) ["Fatigue" in multiple sclerosis. Development and and validation of the "Wurzburger Fatigue Inventory for MS"]. Nervenarzt 77: 165-166, 168-170, 172-164.

2. Beck AT, Ward CH, Mendelson M, Mock J, Erbaugh J (1961) An inventory for measuring depression. Arch Gen Psychiatry 4: 561-571.

3. Derogatis LR, Rickels K, Rock AF (1976) The SCL-90 and the MMPI: a step in the validation of a new self-report scale. Br J Psychiatry 128: 280-289.

4. Hoppe CD, Muller UD, Werheid KD, Thone AD, von Cramon YD (2000) Digit Ordering Test: clinical, psychometric, and experimental evaluation of a verbal working memory test. Clin Neuropsychol 14: 38-55.

5. Arbuthnott K, Frank J (2000) Trail making test, part B as a measure of executive control: validation using a set-switching paradigm. J Clin Exp Neuropsychol 22: 518-528.

6. Corrigan JD, Hinkeldey NS (1987) Relationships between parts A and B of the Trail Making Test. J Clin Psychol 43: 402-409.

7. Gaudino EA, Geisler MW, Squires NK (1995) Construct validity in the Trail Making Test: what makes Part B harder? J Clin Exp Neuropsychol 17: 529-535.

8. Aschenbrenner S, O T, K L (2000) Regensburger Wortflüssigkeitstest. Lisse: Swets und Zeitlinger Verlag.

9. Ashburner J (2007) A fast diffeomorphic image registration algorithm. Neuroimage 38: 95-113.

10. Genovese CR, Lazar NA, Nichols T (2002) Thresholding of statistical maps in functional neuroimaging using the false discovery rate. Neuroimage 15: 870-878.

11. Jenkinson M, Beckmann CF, Behrens TE, Woolrich MW, Smith SM (2012) Fsl. Neuroimage 62: 782-790.

12. Smith SM, Jenkinson M, Johansen-Berg H, Rueckert D, Nichols TE, Mackay CE, et al. (2006) Tract-based spatial statistics: voxelwise analysis of multi-subject diffusion data. Neuroimage 31: 1487-1505.

13. Smith SM, Nichols TE (2009) Threshold-free cluster enhancement: addressing problems of smoothing, threshold dependence and localisation in cluster inference. Neuroimage 44: 83-98.
